# Heterochiasmy and Sex Chromosome Evolution in *Silene*

**DOI:** 10.3390/genes14030543

**Published:** 2023-02-22

**Authors:** Dmitry A. Filatov

**Affiliations:** Department of Biology, University of Oxford, South Parks Road, Oxford OX1 3RB, UK; dmitry.filatov@biology.ox.ac.uk

**Keywords:** sex chromosome evolution, heterochiasmy, genetic mapping, *Silene latifolia*

## Abstract

The evolution of a non-recombining sex-specific region is a key step in sex chromosome evolution. Suppression of recombination between the (proto-) X- and Y-chromosomes in male meiosis creates a non-recombining Y-linked region (NRY), while the X-chromosome continues to recombine in females. Lack of recombination in the NRY defines its main properties—genetic degeneration and accumulation of repetitive DNA, making X and Y chromosomes very different from each other. How and why recombination suppression on sex chromosomes evolves remains controversial. A strong difference in recombination rates between the sexes (heterochiasmy) can facilitate or even cause recombination suppression. In the extreme case—complete lack of recombination in the heterogametic sex (achiasmy)—the entire sex-specific chromosome is automatically non-recombining. In this study, I analyse sex-specific recombination rates in a dioecious plant *Silene latifolia* (*Caryophyllaceae*), which evolved separate sexes and sex chromosomes ~11 million years ago. I reconstruct high-density RNAseq-based genetic maps including over five thousand genic markers for the two sexes separately. The comparison of the male and female maps reveals only modest heterochiasmy across the genome, with the exception of the sex chromosomes, where recombination is suppressed in males. This indicates that heterochiasmy likely played only a minor, if any, role in NRY evolution in *S. latifolia*, as recombination suppression is specific to NRY rather than to the entire genome in males. Other mechanisms such as structural rearrangements and/or epigenetic modifications were likely involved, and comparative genome analysis and genetic mapping in multiple Silene species will help to shed light on the mechanism(s) of recombination suppression that led to the evolution of sex chromosomes.

## 1. Introduction

Recombination (or lack of it) is one of the most important factors affecting evolutionary change [1,2]. The power of recombination to shape the properties of the genome is well illustrated by the contrasting properties of non-recombining regions on Y-chromosomes (NRY) and recombining X-chromosomes (or W- and Z-chromosomes in female heterogamety). Despite originating from a single pair of autosomes and initially having the same gene composition [3], their properties are very different. While the X-chromosomes are full of genes, the NRYs are typically genetically degenerate—they are poor in genes and rich in repetitive DNA [4]. It is reasonably well understood why suppression of recombination leads to genetic degeneration [5,6,7], but it is less clear how and why recombination suppression evolves on nascent sex chromosomes [8,9,10]. This paper focuses on the possible role of heterochiasmy in recombination suppression in NRY during the evolution of sex chromosomes.

The evolution of sex chromosomes is arguably one of the most significant genomic changes that lead to wide-reaching consequences throughout the genome [4,11]. Previous work in mammals, *Drosophila* and plants shed light on many aspects of sex chromosome evolution [4,11]. It is clear that sex chromosomes typically evolve from autosome(s) that acquire sex-determining gene(s), stop recombining around that gene in the heterogametic sex [3] and gradually degenerate afterward [4,12]. This process can be repeated multiple times in the history of a species via the evolution of neo-sex-chromosomes due to translocations between sex chromosomes and autosomes [13,14], or via sex chromosome turnover events [15], with sex-determining gene(s) moving between the chromosomes, restarting the process of recombination suppression and Y(or W) degeneration in a new location in the genome. For example, 13 sex chromosome turnover events occurred in the last ~60 million years across 28 true frog species [16], and multiple turnover events were reported in *Diptera* [17] and cichlids [18].

Despite the progress in our understanding of sex chromosome evolution, many pivotal questions remain unanswered [4]. In particular, how nascent sex chromosomes originate in the first place and how recombination suppression between them evolves remains poorly understood [9,10,19], not least because the model organisms, such as humans and *Drosophila*, have relatively old sex chromosomes that tell us little about the early steps of sex chromosome evolution [4,8]. While studies of neo-sex chromosomes and turnovers of sex chromosomes in animals have yielded valuable information [6,17,20], the initial steps of de novo sex chromosome evolution remain poorly studied. Plants have highly diverse mating systems [21], ranging from hermaphrodites to separate sexes (dioecy). The evolution of dioecy, accompanied by the evolution of sex chromosomes can occur repeatedly within the same plant genus [22]. For example, sex chromosomes evolved de novo at least three times independently in the plant genus *Silene*—in *S. latifolia*, which is the focus of this study, and in *Silene otites* and *Silene pseudotites* [23], which belong to a different section of this large, primarily non-dioecious, genus. Repeated evolution of separate sexes and sex chromosomes in plants offer an opportunity to study how sex chromosomes originate de novo when a species evolves separate sexes.

The cessation of recombination in the sex-specific region on the Y(or W)-chromosome is a key step in sex chromosome evolution, but how recombination suppression evolves is not well understood [8,9,10]. Studies in many different organisms indicated that the non-recombining sex-specific region tends to expand over time, including a larger proportion of the sex chromosome. These expansions leave a characteristic signature of ‘evolutionary strata’—lower divergence between the X and Y (or Z and W) chromosomes in regions that stopped recombining more recently (‘younger strata’) compared to older non-recombining regions (‘older strata’) [24]. Such stratification of divergence between the X and Y (or Z and W) chromosomes was reported in many organisms that evolved sex chromosomes independently from each other (e.g., [24,25,26]). Why such expansions of the NRY occur remains unclear and is actively discussed in the literature [9,19,27,28]. Sexually antagonistic (SA) genes are thought to play an important role in this process [29,30,31], though relatively little experimental evidence in support of this hypothesis is available [32]. Alternatives to the SA hypothesis, proposed to explain expansion of the NRY, include early emergence of dosage compensation [28], neutral divergence between the X- and Y-chromosomes [27,33,34,35], sheltering of deleterious mutations by permanent heterozygosity in males [36,37,38] and heterochiasmy [39,40].

It has been suggested that the difference in recombination rates between the sexes (heterochiasmy) can significantly facilitate the evolution of a non-recombining sex-specific region [39,40]. In particular, the recombination rate in the sex-linked region of a chromosome is dependent on the recombination in that sex. In the extreme case, the lack of recombination in the heterogametic sex, the entire chromosome bearing the sex-determining gene will lack recombination. For example, in *Diptera* (e.g., *Drosophila*) recombination is restricted to females, while in *Lepidoptera* only males recombine, resulting in a lack of recombination on dipteran Y- and lepidopteran W-chromosomes. In less extreme cases, reduced (but present) recombination in the heterogametic sex may facilitate the expansion of the NRY due to stronger linkage disequilibrium between the SA loci and the sex-determining region [30,41]. The distribution of recombination along the chromosomes can differ between the sexes, with the tendency for tip-biased recombination in male meiosis and more uniform distribution in females [41]. While limited data are available for plants, differences in recombination rates between sexes were reported for hermaphroditic *Arabidopsis thaliana* [42], *Brassica nigra* [43], *Hordeum vulgare* [44] and dioecious *Rumex hastatulus* [39]. In the latter case, the heterochiasmy was suggested to have played a significant role in the evolution of *Rumex* sex chromosomes. In this study, I report the analysis of sex-specific recombination rates in another dioecious plant species that evolved sex chromosomes quite recently—about 11 million years ago [45]—*S. latifolia* (*Caryophyllaceae*).

White campion (*S. latifolia*, previously known as *Silene alba* and *Melandrium album*), was the first plant where sex chromosomes were discovered—exactly 100 years ago [46], and there is a long history of cytogenetic and genetic studies of sex chromosomes in this species [47,48]. Not dissimilar to mammals and birds, sex chromosomes in *S. latifolia* are highly heteromorphic, though, unlike mammals and birds (where Y or W are typically small), the Y-chromosome in *S. latifolia* is the largest and the X is the second largest in the genome (e.g., [49]). The presence of highly distinct heteromorphic sex chromosomes in *S. latifolia* contrasts with many other dioecious plant species studied so far, such as papaya [50], persimmon [51], kiwifruit [52], asparagus [53], ginkgo [54] and *Mercurialis* [55], where sex chromosomes are cytologically indistinguishable (homomorphic) and have only small NRY that is flanked by long pseudoautosomal regions (PARs).

Genus *Silene* includes over 500 species [56] most of which are non-dioecious [57], and dioecy is clearly a derived trait in *S. latifolia* and its close relatives [58,59]. According to evolutionary genetic analyses based on sequence divergence and estimates of mutation rate in *S. latifolia*, sex chromosomes in this lineage have evolved 11 (95% CI: 7.83–15.03) million years ago [45]. The age of sex chromosomes roughly corresponds to the time of *S. latifolia* divergence from non-dioecious *Silene* species, suggesting that separate sexes and sex chromosomes evolved simultaneously or nearly simultaneously [58]. Comparative genetic mapping in *S. latifolia* and its non-dioecious relatives revealed that sex chromosomes likely evolved from a single pair of chromosomes [60]. Deletion mapping of the Y-chromosome revealed the location of two Y-linked sex-determining genes—the stamen promotion factor (SPF) and gynoecium suppression factor (GSF) [48,61], the latter of which was recently isolated and characterized molecularly [62]. Relatively recent de novo evolution of heteromorphic sex chromosomes in *S. latifolia* offers an opportunity to study the origination of sex chromosomes rather than their turnover, which is often analysed in animal literature [15,17]. In this paper, I analyse recombination rates in *S. latifolia* male and female meiosis and test whether heterochiasmy could have played a role in the sex chromosome evolution of this species.

## 2. Materials and Methods

The sex-specific genetic maps were reconstructed using the transcriptome sequence data from Papadopulos et al., 2015 [63]. The mapping family included grandparents and parents, as well as 20 males and 32 females of the F2 progeny. The grandparents of the mapping cross were grown from seed collected in the wild, with the female and male plants originating in Salzburg (Austria) and Oxford (UK), respectively. Raw RNAseq reads from grandparents, parents and 52 progeny generated by Papadopulos et al., 2015 [63], were mapped to the published [64] female *S. latifolia* transcriptome including 19,195 cDNA contigs—the same reference sequence as used in the previous genetic mapping [63]. Paired end RNAseq reads trimmed with Trimmomatic [65] were mapped against the reference transcriptome with BWA mem 0.7.17 [66] and sorted with Samtools 1.7 [67]. Then, single nucleotide polymorphism (SNP) calls were generated with Samtools mpileup (options: -d 1000 -q 20 -Q 20), and sites were filtered with bcftools filter 1.7. The resulting vcf file including multiple individuals was filtered to exclude any SNPs with quality lower than 999. To generate female and male-specific maps the SNPs were split into male- and female-informative SNPs using an awk one-line script: “awk ‘{if (($10~“0/1”)&&($11~“0/0”)) print}’ allSNPs.vcf >maleInformativeSNPs.vcf” and “awk ‘{if (($10~“0/0”)&&($11~“0/1”)) print}’ allSNPs.vcf >femaleInformativeSNPs.vcf”, where the fields “$10” and “$11” correspond to SNP calls in the paternal and maternal individuals, respectively. The resulting female- and male-informative SNPs were used to reconstruct sex-specific maps with lepMap3 [68]. All the steps of running the lepMap3 were automated in the script runLepMap3.sh that converted SNP calls in the VCF file to the ‘posterior’ lepMap format and consecutively ran ParentCall2, Filtering2 (with dataTolerance = 0.001), SeparateChromosomes2 (with lodLimit = 5), JoinSingles2All (with parameters lodLimit = 3 lodDifference = 2) and OrderMarkers2 (with recombination1 = 0 or recombination2 = 0 for the female and male maps, respectively). The resulting SNP-level maps were loaded into excel and semi-manually filtered to exclude any cDNA contigs with different SNPs giving different locations in the genetic map.

## 3. Results

Previous genetic mapping work in *S. latifolia* has yielded a sex-averaged genetic map including 2114 genic markers, 327 of which were located on the X-chromosome (including 108 pseudoautosomal markers) [63]. That map was based on the analysis of the segregation of SNPs detected in transcriptome sequence data from parents and 52 progeny in a genetic cross. Here, I re-use RNA-seq data from that study to construct and compare sex-specific genetic maps. To facilitate the comparisons with the previous work I use the same reference transcriptome from [64], including 19,195 cDNA contigs used in the previous genetic mapping [63]. Sequence read mapping to that reference, followed by SNP calling and filtering (see methods) has yielded 31,015 and 24,502 high-quality SNPs informative for male and female maps, respectively. The high-quality SNPs were used for the reconstruction of sex-specific genetic maps with Lep-Map3 [68], as described in the methods. The resulting sex-specific maps included 12 linkage groups (LGs; Tables S1 and S2), corresponding to the number of chromosomes in *S. latifolia*. The female and male maps were 928.26 and 1004.45 cM long, respectively (Table 1). This is similar to the length of the previously constructed sex-average map (1017.08 cM [63]). The number of markers per LG ranged from 204 to 872 and the total number of mapped genes was 5647 and 5535 in the female and male maps, respectively (Table 1). This nearly triples the number of mapped genes compared to the previously published sex-average map [63].

**Figure 1:**
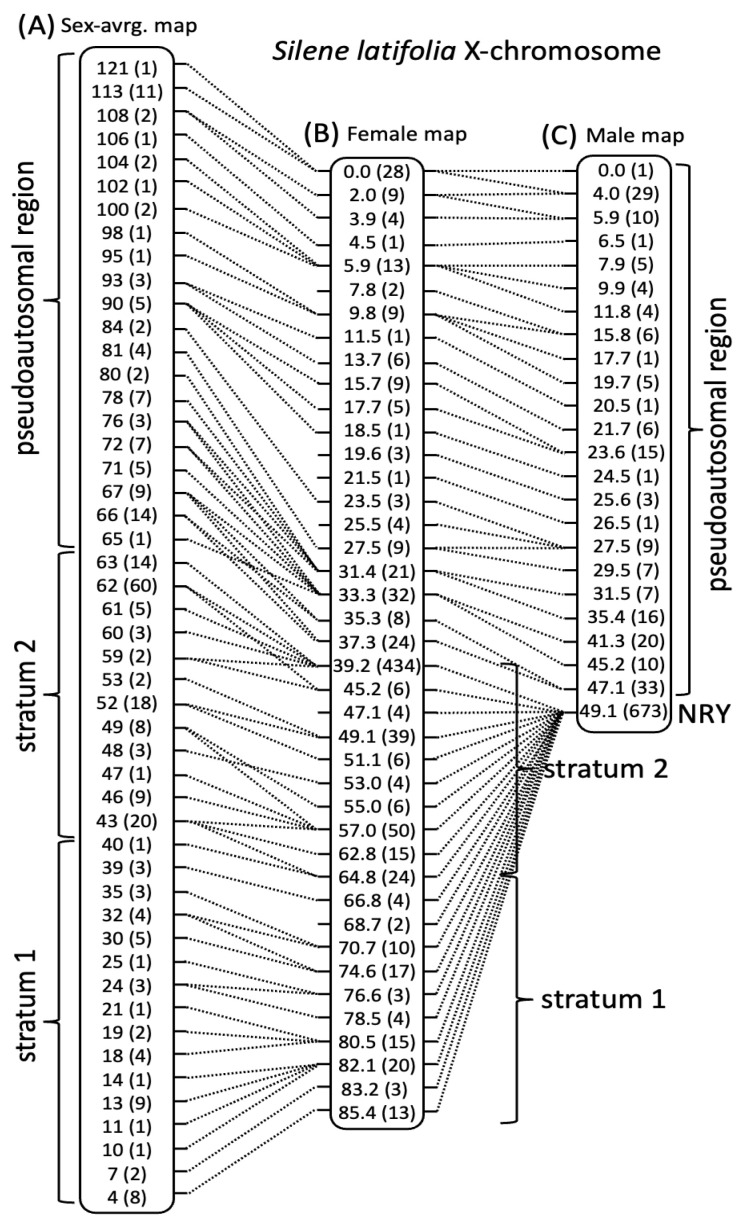
Comparison of genetic maps for the X-chromosome of *S. latifolia*. (**A**) Sex-average map showing only the markers shared with the female map constructed in this study (**B**). (**C**) Male genetic map. The genetic distances are shown for each position (in cM), rounded to integer values in (**A**). The numbers of genes sharing the same or very similar position are shown in brackets. The genetic positions sharing genic markers in different maps are connected by dotted lines. The locations of the pseudoautosomal region (PAR), the non-recombining Y-linked region (NRY) and two evolutionary strata are shown.

According to the genes shared by the LGs in different maps, each LG in the newly constructed sex-specific maps had an unambiguous one-to-one correspondence to an LG in the sex-averaged map [63]. In particular, the largest (by the number of markers) LG included 872 genes, 279 (including 84 pseudoautosomal genes) of which represented previously identified sex-linked genes present on the X-chromosome in the previously published map [63]. None of the other LGs included any previously identified sex-linked genes. Thus, the largest linkage group in the two sex-specific maps likely corresponds to the X-chromosome. Consistent with X-linkage, and recombination suppression between the X- and Y-chromosomes in males, the largest LG in the female map was much longer (85.4 cM) compared to the corresponding LG in the male map (49.1 cM). Furthermore, the distribution of recombination along these LGs is consistent with X-linkage (Figure 1). In particular, the genetic lengths of the region containing previously identified 84 pseudoautosomal genes was comparable in the male and the female maps (47.1 and 37.3 cM, respectively), while the region containing X-linked genes is collapsed in the male map (0 cM), but not in the female map (46.1 cM). Given the one-to-one correspondence of the LGs in different maps, the names of the LGs in the previously published map were used for the corresponding LGs in the newly constructed sex-specific maps (Table 1) to ensure uniformity and simplify comparisons between the maps.

**Figure 2:**
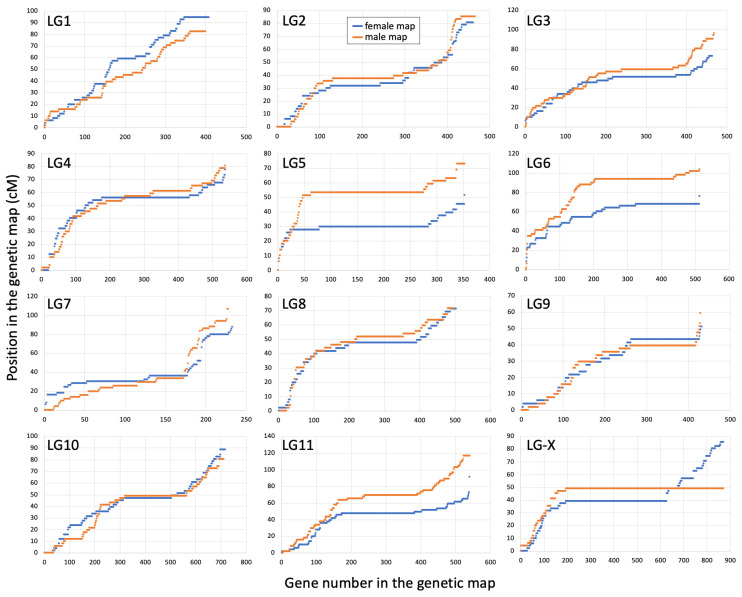
Cumulative genetic distance for 12 linkage groups (LG) in the female (blue) and male (orange) genetic maps.

The other linkage groups (excluding the X) range in size from 218 to 673 genes in the female map and from 204 to 659 genes in the male map (Table 1). The length of the autosomal LGs ranged from 51.1 to 94.6 cM in the female map and from 59.2 to 116.6 cM in the male map. The distribution of recombination along the LGs was quite uneven in both sexes, with most recombination occurring closer to the tips of the chromosomes. LG1 was an exception to this tip-biased distribution of recombination, with relatively even recombination along its length (Figure 2). The reason for this peculiarity of the LG1 is not clear. The length of LGs and distribution of recombination along the LGs is similar in the two sexes for most autosomes, except LGs 5, 6 and 11, where the male maps were somewhat longer compared to the female maps (Table 1 and Figure 2), revealing relatively modest heterochiasmy in *S. latifolia*. The difference between the sexes for the LGs 5, 6 and 11 is mostly due to slightly lower recombination at one or both ends of the chromosomes in females compared to males.

## 4. Discussion

**Figure 3:**
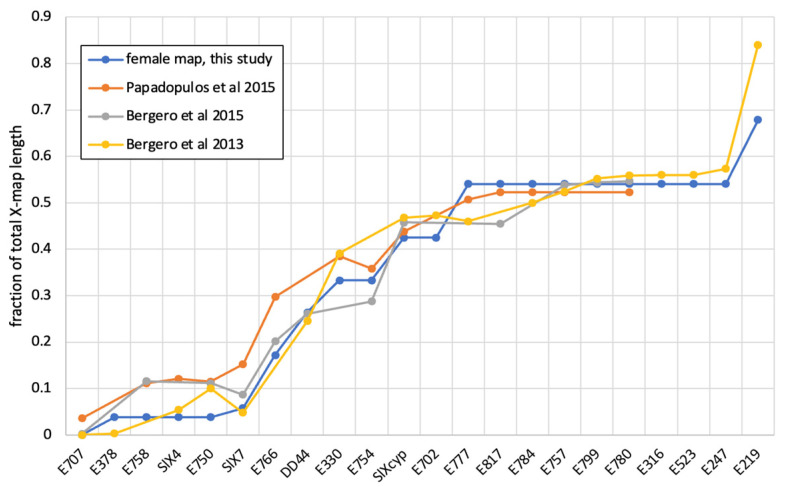
A comparison of different genetic maps for the *S. latifolia* X-chromosome. Names of the genes are shown along the horizontal axis. The vertical axis shows a rescaled fraction of the total map length for each gene. The genetic positions for *S. latifolia* genes listed in Table 2 were rescaled and re-oriented for maps to have the same orientation and length. The male map is not shown because it lacks recombination [62,69,70].

The main result of this paper is the construction of high-density sex-specific genetic maps for *S. latifolia*. While our genetic mapping is based on a single genetic family [63], the comparison with the maps in other studies [69,70] reveals a good correspondence between independent genetic maps of the *S. latifolia* (Figure 3), although only a few markers are shared by these maps (Table 2). 

These high-density sex-specific maps will be instrumental in the future genomic and evolutionary genetic analyses involving *S. latifolia* genome once it becomes available. The previously published *S. latifolia* genome assembly [63] is too fragmented to be integrated with the genetic map, with most genomic contigs containing one or no genetic markers. In the absence of the chromosome-level genome assembly, the genetic maps provide spatial information about the location of the particular genes in the genome, which is important for many analyses, including the tests for selection [71] and the inference of introgression of genetic material between species [72,73]. For example, the genetic map-based spatial information along the chromosomes was used in recent analyses of sex chromosome evolution [74,75] and in the studies of speciation [76,77] in *S. latifolia* and its close relatives. The high-density maps (with >5500 genes mapped) reported here, will facilitate the work in this interesting species that served as a model system for many topics in ecology and evolution [78].

The newly constructed maps nearly triple the number of mapped genes compared to the previously published [63] sex-averaged map (Table 1). The gene order and genetic distances are quite similar in the three maps, except for the X-chromosome where the male map is much shorter due to the suppression of recombination in the NRY. Despite this, the overall male map is 76 cM longer compared to the female map, with most linkage groups slightly longer in the male map or similar in both sexes (Table 1). This indicates slightly more frequent recombination in male compared to female meiosis in *S. latifolia*. 

The sex-averaged map [63] is longer than both the male and female maps, though the difference is quite small (Table 1), except for the X-chromosome, where the sex-average map is 30% longer than the female map (Figure 1). The larger difference in the X-chromosome could, at least partly, be due to the pseudoautosomal region (PAR) that is 50% longer in the sex-average compared to the female map. The PAR of the X-chromosome is ~20% longer in the male map compared to the female map (Figure 1), which is consistent with the general tendency for elevated recombination in PAR in males due to the clustering of chiasmata in this region to ensure X:Y pairing in male meiosis [79]. However, the sex difference in PAR map lengths in *S. latifolia* is relatively modest and far smaller than in humans, where the recombination rate in the PAR1 in males is at least an order of magnitude higher compared to females [80]. Due to the requirement of at least one crossover per meiosis for proper chromosome segregation, recombination density in the PAR in males is expected to be negatively proportional to PAR size [81]. Only a modest difference in male and female map lengths in *S. latifolia* PAR (Figure 1) indicates that this region is likely physically large.

The comparison of the newly constructed genetic maps with the previously published map of non-dioecious *Silene vulgaris* [69] revealed the differences in gene order in the homologous chromosomes of the two species (Table 2). This likely reflects structural rearrangements, with inversions and translocations changing the gene order in the maps of these two species. It is interesting to speculate that at least some of these rearrangements were involved in recombination suppression and NRY evolution on *S. latifolia* sex chromosomes. However, the genetic map of *S. vulgaris* is too sparse for more definitive conclusions. Furthermore, without information from an outgroup species, it is not possible to tell whether the rearrangements occurred in *S. vulgaris* or in *S. latifolia* lineage after these species diverged. Thus, the comparisons of high-density genetic maps from multiple *Silene* species are needed to infer the sequence of events that led to NRY formation and expansion in *S. latifolia*. These analyses, supplemented by a high-quality reference sequence for at least one of the *Silene* species, will be very informative about the history of sex chromosome evolution.

Another important finding of this work is the non-random tip-biased distribution of recombination on most chromosomes (Figure 2). However, without the comparison of the genetic maps to the genome sequence, it is not possible to infer the actual size of the actively and rarely recombining regions. In particular, local variations in gene density along the chromosomes could, at least partly, account for the observed non-evenness in the distribution of recombination along the LGs. The tip-biased distribution of recombination is widespread in plants, though not universal [82], with the peripheral distribution of recombination often present in species with large chromosomes (>100 Mb) [82,83]. The causes of this bias are not entirely clear, but it could be driven by mechanistic constraints as postulated by the ‘telomere-initiation’ model [83,84]. Alternatively, this pattern could be caused by adaptation-related factors. For example, it is possible to speculate that tip-biased distribution of recombination is advantageous as it ensures a sufficient recombination rate in gene-rich regions often located closer to the ends of the chromosomes [82].

Finally, the analysis of heterochiasmy in *S. latifolia* and its possible role in sex chromosome evolution was the main motivation for this work. Interestingly, the analysis presented above revealed very modest heterochiasmy in *S. latifolia*, which contrasts with the recent reports of a much stronger difference in recombination between the sexes in guppy [40] and dioecious plant *R. hastatulus* [39], where heterochiasmy is proposed to play an important role in sex chromosome evolution. As expected, the strongest difference in recombination between the sexes in *S. latifolia* was observed on the X-chromosome, which recombines along its length in females and only at one end—in the PAR, in males (Figure 1C). Modest heterochiasmy was apparent on LGs 5, 6 and 11 (Figure 2), though the direction of difference in recombination rate between the sexes was the opposite of that expected for heterochiasmy to facilitate sex chromosome evolution with male heterogamety. In particular, the idea of heterochiasmy promoting the formation and expansion of the NRY is that the recombination in the heterogametic sex is reduced compared to the homogametic sex, which helps to increase the size of the region linked or partly linked to the sex locus. However, the difference in recombination rates between the sexes observed on LGs 5, 6 and 11 is in the opposite direction—more recombination in the heterogametic than homogametic sex (Figure 2), making the overall length of the male map slightly longer compared to the female map (Table 1). Thus, although modest heterochiasmy is present in *S. latifolia*, it is unlikely to promote the evolution of the NRY in this male heterogametic species. If heterochiasmy in other *Silene* species is in the same direction as in *S. latifolia*, it would help the evolution of ZW sex chromosomes with female heterogamety, as reported in *Silene otites* [23].

## Figures and Tables

**Table 1:** The comparison of female, male and sex-average *S. latifolia* genetic maps.

	Female Map		Male Map		Sex-Average Map	
LGs	Genes	Length	Genes	Length	Genes	Length
LG1	385	94.61	373	82.36	219	92.29
LG2	416	80.64	432	85.05	207	91.24
LG3	428	73.05	426	96.33	185	99.94
LG4	488	77.48	507	80.73	163	81.35
LG5	332	51.64	339	73.05	165	68.70
LG6	479	75.86	407	103.64	169	75.71
LG7	218	87.71	204	106.53	152	86.93
LG8	469	71.17	413	71.42	142	79.61
LG9	410	51.07	402	59.16	143	53.95
LG10	673	88.41	659	80.48	134	98.14
LG11	477	91.27	505	116.59	108	67.97
X	872	85.35	868	49.11	327	121.25
All	5647	928.26	5535	1004.45	2114	1017.08

**Table 2:** Genetic positions of *S. latifolia* X-linked genes and their *S. vulgaris* homologs.

Gene Name		*S. lat.* (This Study)		*S. lat.* Sex-Avrg. Maps			*S. vulgaris* LG12	
This Study	[69,70]	Female	Male	[63]	[69]	[70]	[69]	[70]
Contig4232	E707	85.348	49.106	4.26	0.2	0	32.1	29.7
Contig18305	E378	82.113	49.106		0	0.3	28.8	26.9
Contig13157	E758	82.113	49.106	13.36	10.4		45.7	
Contig8519	SlX4	82.113	49.106	14.49		5.4		
Contig9453	E750	82.113	49.106	13.74	10	10	31.5	
Contig842	SlX7	80.483	49.106	18.22	7.8	4.8	41.5	40.1
Contig4853	E766	70.678	49.106	35.7	18.1		38.4	
Contig1807	DD44	62.834	49.106		23.5	24.5	36.8	37.4
Contig4971	E330	56.951	49.106	46.14		39.1		
Contig2851	E754	56.951	49.106	42.92	25.9		37.1	
Contig8805	SlXcyp	49.106	49.106	52.55	41.2	46.8	47.9	47.5
Contig4251	E702	49.106	49.106			47.3		40.9
Contig3001	E777	39.221	49.106	60.88		46		
Contig1564	E817	39.221	49.106	62.69	40.9		51.8	
Contig9553	E784	39.221	49.106	62.69		50		
Contig255	E757	39.221	49.106	62.72	48.4	52.4	18.7	17.6
Contig9591	E799	39.221	49.106		48.9	55.2	24.7	23.6
Contig8488	E780	39.221	49.106	62.69	49.2	55.9	14.8	12.4
Contig4305	E316	39.221	49.106			56		
Contig18190	E523	39.221	49.106			56		
Contig9077	E247	39.221	49.106			57.3		
Contig17205	E219	27.455	29.496			84		

## Data Availability

No new data was generated in this study.

## Supplementary Materials

The following supporting information can be downloaded at: https://www.mdpi.com/article/10.3390/genes14030543/s1, Table S1: Female map; Table S2: Male map.
